# A Task Force Study on Community-Level Situational Analysis in Urban and Rural Bhopal: Assessing the Availability and Acceptability for Doorstep Health-Care Services

**DOI:** 10.7759/cureus.97295

**Published:** 2025-11-19

**Authors:** Devendra Gour, Manju Toppo, Jugal Kishore, Neeta Kumar, Rama S Lodha, Seema Patel, Khushali Solanki, Khushboo Gupta, Paramjeet S Mewara, Anurag Bharti

**Affiliations:** 1 Community Medicine, Gandhi Medical College, Bhopal, IND; 2 Community Medicine, Vardhman Mahavir Medical College and Safdarjung Hospital, New Delhi, IND; 3 Research, Indian Council of Medical Research, New Delhi, IND

**Keywords:** abha id, community health, diagnostic services, doorstep health care, icmr task force, universal health coverage

## Abstract

Background

Equitable access to health care remains a persistent challenge in India, particularly among underprivileged urban and rural populations. Despite initiatives like Ayushman Bharat and Health and Wellness Centres, disparities in awareness, accessibility, and affordability persist. This study, conducted as part of the Indian Council of Medical Research (ICMR) Task Force project, aimed to assess the availability, awareness, and acceptability of doorstep health-care and diagnostic services in Bhopal, Madhya Pradesh.

Methods

A community-based cross-sectional survey was conducted from November 2022 to May 2023 among 5,000 individuals (2,500 each from rural and urban sites) representing socioeconomically disadvantaged populations. Study blocks were selected from the enlisted blocks by the probability proportional to size (PPS) method. Data were collected using a pre-validated questionnaire covering health-care awareness, service utilisation, preferences, and digital health identity status through the Ayushman Bharat Health Account status (ABHA) ID. The statistical analysis was performed using Epi Info 7 (Centers for Disease Control and Prevention, Atlanta, GA).

Results

Awareness of existing health-care facilities was moderate (urban: 49.6%; rural: 45.0%), with most respondents identifying primary health centres (PHC)/Wellness Centres and community health centres (CHC). Allopathy was the preferred system of care (>97%), and government doctors were favoured by 76.1% of urban and 54.5% of rural participants. Diagnostic service utilisation was extremely low, with over 97% reporting no tests in the last three months. Acceptability for doorstep diagnostic services was high and consistent across both urban (66.8%) and rural (66.6%) sites. Only 4.1% of participants possessed an ABHA ID, reflecting limited digital health integration. The main barriers included lack of awareness (17.3%) and unlinked Aadhaar-mobile connections (46.9%)(Aadhaar number is a 12-digit random number issued by the Unique Identification Authority of India (UIDAI) ('Authority') to the residents of India after satisfying the verification process laid down by the Authority).

Conclusion

The study highlights significant gaps in health-care awareness, diagnostic access, and digital health adoption among disadvantaged communities. However, the high acceptability of doorstep services offers a promising avenue for improving early diagnosis and continuity of care. Strategic implementation of mobile diagnostic technologies, targeted community awareness, and strengthening of frontline health workers are essential to bridge these gaps and advance towards universal health coverage (UHC).

## Introduction

Primary health care employs an integrated approach that combines preventive, promotive, curative, and rehabilitative services for individuals, families, and communities. Primary health care is the point of entry for individuals into the national health system. The continuous availability of good-quality curative services satisfies people and motivates the community to engage in preventive and promotive services. High reporting for healthcare is, however, a positive indication, as it reflects the health-seeking behaviour of the people. Turning high out-of-pocket expenditure (OOPE) payment for health care at the same time is directly responsible for the increase in overall poverty [1].

The National Health Policy 2017 emphasises equitable access to health services, particularly for vulnerable and marginalised groups. Despite initiatives such as the National Rural Health Mission (NRHM) and Ayushman Bharat Health and Wellness Centres, equitable access to quality health care and low awareness among the community regarding these health schemes remain a challenge in India. With the rising burden of both communicable and non-communicable diseases, timely access to diagnostic facilities and primary care at the community level has become essential. However, limited availability of laboratories, shortage of trained personnel, poor referral linkages, and weak community-based service delivery often force underprivileged populations to depend on informal providers or delay care-seeking, exacerbating disease burden [2].

India is suffering from inadequate health facilities, including a shortage of medicine/drugs, equipment, basic amenities, health staff in hospitals, and primary health centres (PHCs). The cost and poor quality of services, as well as discriminatory treatment by health professionals, disproportionately hinder disadvantaged groups from taking up community health worker (CHW) referrals. A variety of situation analysis studies from Karnataka and Gujarat observed the same gaps and requirements of labs, manpower, and networking at the grassroots [3,4]. India’s situation for workforce and infrastructure was reviewed by Garg et al. [5] and Iyengar et al. [6] in Bihar for the availability of workforce, drug supply [7], and doctors in rural areas observed a dissatisfactory situation [8]. Hence, this task force study is to include study areas from Gujarat, Bihar, North East, Uttar Pradesh, and one from the south and north.

Ayushman Bharat Scheme impact assessment studies found that enrolment in public health insurance programs for the poor increased the utilisation of inpatient health care [9]. However, overuse and fraud were also reported. Achieving Universal Health Coverage (UHC) is an important goal for almost every nation in the world [10-12], but only a few have worked towards its logical implementation [13]. In Indian circumstances, indigenous, locally, culturally appropriate methods are required [1,14-17]. The National Health Policy (NHP) and the National Digital Health Mission (NDHM) document gave the road map policy [18]. Now efforts are required to place necessary connections, communication, channels, routes, manpower, and their skilling to implement all envisaged targets under Health for All-NDHM.

Improved access to health care and the reduction of the overall burden of OOPE, especially in the most vulnerable sections of the population, are likely to require additional interventions that address gaps in the availability of care and provide patients appropriate pathways that support their journey from a strong and comprehensive primary care service where they may be informed of their entitlements and investigated for their initial symptoms to appropriate hospitals for the treatment of serious illness.

Ayushman Bharat Health Account status (ABHA) ID is a milestone step towards promoting and achieving the goal of telehealth by providing health-care providers with access to patient medical records, enabling them to provide remote consultations and treatments. Thus, the facility will help in bridging the health gaps, especially with regard to the rural, remote, and underprivileged sections of the population of the country, who do not get proper access to health services due to inherent physical and economic challenges [19].

Access to timely and affordable health care is a cornerstone of achieving UHC. Some communities are disproportionately affected by communicable diseases, maternal and child health problems, and nutritional deficiencies, yet their access to diagnostic facilities and doorstep health care remains inadequate.

A situational analysis of the availability and accessibility of medical diagnostic facilities and doorstep health services among underprivileged communities is, therefore, crucial. Such evidence will help identify existing gaps and needs, and the acceptability of doorstep services. Keeping this in view, a Task Force study was conducted at seven sites for representation of the community across the country. So, as a representation of centrally located communities, this study was planned at the Bhopal site. 

Objectives

This study aims to perform a situational analysis of the availability of health care and medical diagnostic testing facilities in urban and rural sites of Bhopal, to find out the acceptability and preparedness of doorstep health-care services, to find out the awareness of the existing health schemes and satisfaction regarding availing these schemes, and to assess the availability of ABHA ID among residents of urban and rural sites in Bhopal. 

## Materials and methods

**Figure 1:**
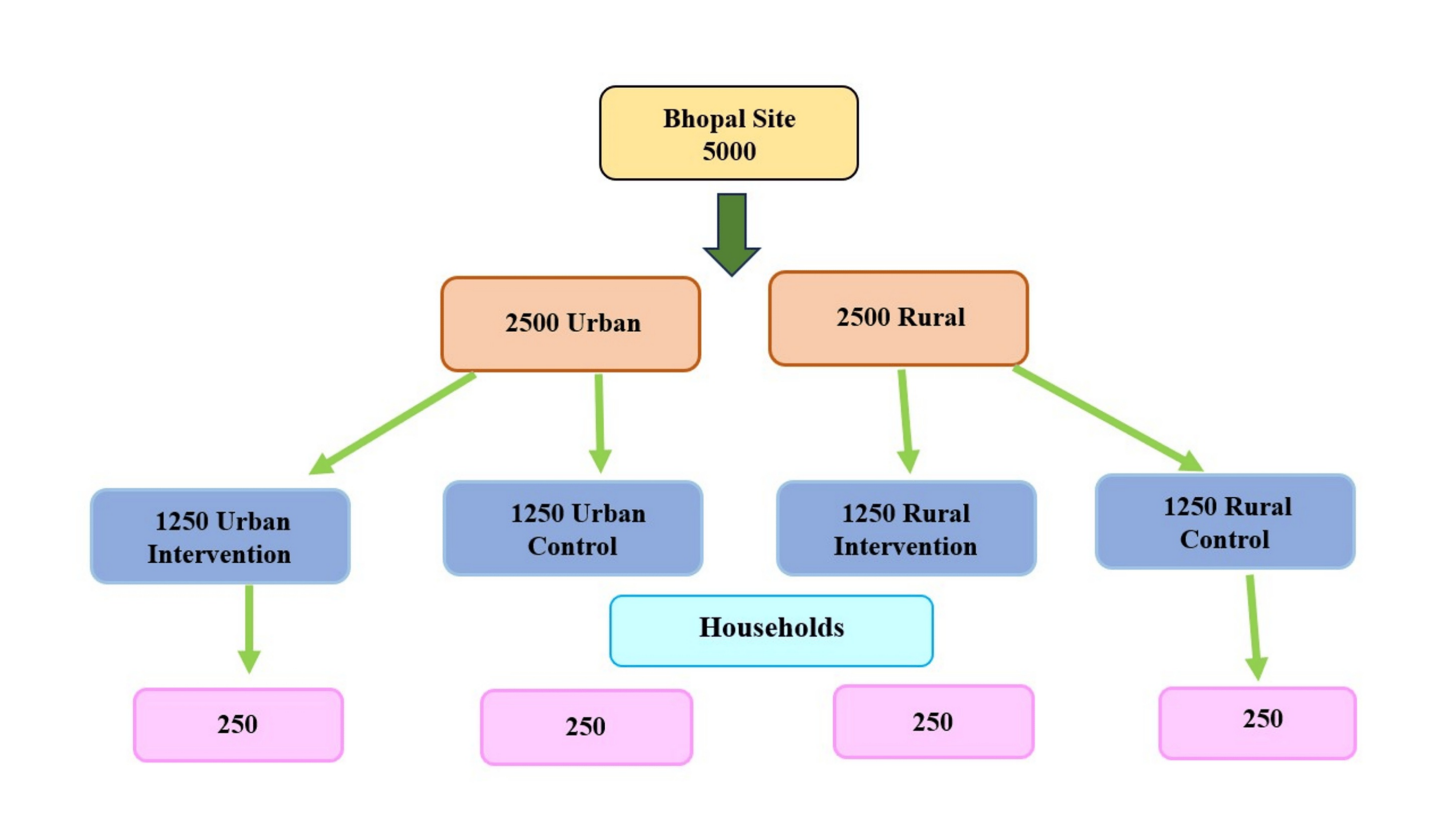
Flowchart of the study plan for Bhopal site

As part of the baseline survey for the multi-centric study under the Indian Council of Medical Research (ICMR) Task Force project titled 'Task Force study for evaluation of community level acceptability, scalability and linkage within the health system of ICMR pre-validated Labike technologies for screening & diagnosis in rural and urban population - An Implementation research,' a door-to-door household survey was conducted among a selected underprivileged population, i.e., socially and economically disadvantaged groups residing in rural areas (Bineka and Maholi) and urban areas (Gondipura and Abbas Nagar) of Bhopal site (Figure 1), who face limited access to quality health-care services. As the main Task Force study is an implementation research with a randomised controlled design in site allocation, one intervention and one control arm have been randomly selected in both rural and urban areas. All family members of participating families were included in the study. The head of the family was contacted to obtain consent after providing detailed information about the study. The study was conducted for the duration of one year, from May 2023 to May 2024. The study was approved by the institutional ethics committee. The survey was conducted using a pre-designed, pre-validated questionnaire.

Sample size

The study participants were required to be chosen from the designated impoverished rural and urban areas of 5000 populations (i.e., 1000 households, assuming an average household size of five individuals), including 2500 for intervention and 2500 for control sites to cover the sample size (Figure 1). The sample size of 2500 per area was calculated in the main Task Force study, 'Task Force study for evaluation of community level acceptability, scalability, and linkage within the health system of ICMR pre-validated Labike technologies for screening & diagnosis in rural and urban population-An Implementation research', using the design of implementation studies for quality improvement programs and derived formulas for sample size in implementation research by Cheung and Duan [20]. ​

Study tool

The survey was conducted using a pre-designed, pre-validated questionnaire.

Data analysis

Data were entered into MS Excel (Microsoft Corporation, Redmond, Washington, United States). The statistical analysis was performed using Epi Info 7 (Centers for Disease Control and Prevention, Atlanta, GA). 

## Results

A total of 1309 households were selected for this study. Almost 63.3% households were from the urban site and 36.7% were from the rural site. The majority (38.9%) were aged 18-36 years, followed by 37% below 18 years of age, while only 1.7% were older than 70 years. Males (51.8%) and females (48.2%) were almost equally represented. The majority of participants lived in nuclear families (54.5%), with the remaining (45.4%) belonging to joint families. Approximately half of the respondents in both settings reported awareness of existing health-care facilities, with 49.6% in urban areas and 45.0% in rural areas. Among those who were aware, PHCs and Wellness Centres were the most frequently cited facilities in rural areas (176 mentions). In contrast, community health centres (CHCs) were most widely known in urban sites (264 mentions). Knowledge of higher-level facilities such as district, secondary, and tertiary hospitals was reported by only a small proportion of participants in either setting, indicating limited awareness of referral-level services (multiple responses were allowed for this question). Regarding the preferred health system, an overwhelming majority of participants in both urban (98.8%) and rural (97.9%) sites expressed a preference for allopathic care. Other systems, such as homoeopathy, ayurveda, unani, and traditional practices, were reported by a very small fraction of respondents, though traditional medicine use was relatively higher in rural areas (3.8%) compared to urban areas (0.5%) (multiple responses were possible). When asked about their choice of health-care providers, government doctors were preferred by 76.1% of urban respondents and 54.5% of rural respondents. In contrast, reliance on private providers was substantially higher in rural areas (42.1%) than in urban ones (17.5%), the reason being that they were easily available and provided fast and low-cost treatment (Table 1). 

**Table 1:** Situational analysis of health-care knowledge, preferences among study participants PHC: primary health centre; CHC: community health centre *Multiple answers were allowed for this question

Indicator	Category/response	Urban n (%) (n = 829)	Rural n (%) (n = 480)
Knowledge of existing health-care facilities	Yes	412 (49.6)	216 (45.0)
	No	417 (50.4)	264 (55.0)
If yes → type of facility known*	PHC/Wellness Centre	152 (18)	176 (37)
	CHC	264 (32)	48 (10)
	District hospital	26 (3)	3 (1)
	Medical college and hospital	12 (1)	5 (1)
Preferred health system*	Allopathy	819 (99)	470 (98)
	Homoeopathy	19 (2)	2 (0.4)
	Ayurvedic	13 (2)	6 (1.3)
	Unani	10 (1)	0
	Traditional	4 (0.5)	18 (3.8)
Preference of doctors	Government doctors	631 (76.1)	262 (54.5)
	Private doctors	145 (17.5)	202 (42.1)
	Both	53 (6.4)	16 (3.3)
Knowledge of testing facilities	All routine blood tests	191 (23.0)	37 (7.7)
	Specific biomarkers/advanced tests	537 (64.7)	424 (88.3)
	X-ray	10 (1.2)	1 (0.2)
	CT scan	1 (0.1)	0 (0.0)
	Not known	90 (10.8)	18 (3.8)

Willingness to share health data was only 45.7% in urban and 42.7% in rural sites, with the majority of them desiring to get screened once a month. Knowledge of available diagnostic testing services also showed notable differences between settings. Most respondents recognised 'specific biomarker or advanced tests' as available diagnostic services, 64.7% in urban and 88.3% in rural areas, while awareness of basic blood tests, X-ray, or CT scan facilities was comparatively low (Table 2).

**Table 2:** Situational analysis of health data sharing, service utilisation, and accessibility ASHA: Accredited Social Health Activist

Indicator	Category/response	Urban n (%) (n = 829)	Rural n (%) (n = 480)
Sharing of health data	Yes	429 (51.7)	301 (62.7)
	No	400 (48.2)	179 (37.2)
If yes → shared with (n = 429 urban, n = 301 rural)	Community worker	15 (3.5)	11 (3.6)
	ASHA	186 (43.4)	144 (47.8)
	Anganwadi worker	228 (53.1)	146 (48.5)
Comfortable sharing one’s own health data	Yes	251 (30.2)	152 (31.6)
	No	578 (69.7)	328 (68.3)
Acceptability of doorstep services	Yes	554 (66.8)	320 (66.6)
	No	275 (33.2)	160 (33.3)
Willingness for a doorstep blood test	Yes	554 (66.8)	320 (66.6)
	No	275 (33.2)	160 (33.3)
Utilisation of diagnostic tests (last 3 months)	None	809 (97.4)	475 (98.8)
	1-2 times	13 (1.6)	4 (0.8)
	3-5 times	5 (0.6)	1 (0.2)
	>5 times	3 (0.4)	1 (0.2)
Distance to nearest lab facility	0-1 km	514 (62.0)	360 (75.0)
	1-5 km	192 (23.2)	34 (7.1)
	>5 km	123 (14.8)	86 (17.9)
Sharing health data will help to get preventive and curative care	Yes	379 (45.7)	205 (42.7)
	No	450 (54.3)	275 (57.3)
How frequently should health guide visit to screen and update health data	Once a month	746 (90)	462 (96.25)
	Twice a month	57 (6.9)	12 (2.5)
	Thrice a month	26 (3.1)	6 (1.25)

Table 3 shows health schemes that are extended to all disadvantaged groups and to all people working in the formal and informal sectors. This, however, is a welcoming step for reducing out-of-pocket health payments, but with appropriate implementation, enrolment, and oversight/regulation are even more important. Among the robust government health schemes, mainly the Ayushman Bharat, Janani Suraksha Yojana (JSY), and Integrated Child Development Services (ICDS) were the most preferred schemes to be adopted by the participants. Both the rural and urban sites availed equally of these three schemes and were satisfied, and very few beneficiaries adopted the Central Government Health Scheme (CGHS), Employees' State Insurance Corporation (ESIC), and Pradhan Mantri Suraksha Bima Yojana (PMSBY).

**Table 3:** Awareness of existing heath schemes and satisfaction levels among urban and rural beneficiaries PMSBY: Pradhan Mantri Suraksha Bima Yojana; CGHS: Central Government Health Scheme; ESIC: Employees' State Insurance Corporation; JSY: Janani Suraksha Yojana; ICDS: Integrated Child Development Services

Satisfaction level	Urban		Rural	
	Satisfied (%)	Dissatisfied (%)	Satisfied (%)	Dissatisfied (%)
Ayushman Bharat n rural-33, n urban-82	69 (84.1)	13 (15.9)	26 (78.7)	7 (21.3)
PMSBY n rural-3, n urban-5	3 (60)	2 (40)	2 (66.6)	1 (33.4)
CGHS n rural-1, n urban-1	1 (100)	0	1 (100)	0
ESIC n rural- 0, n urban-4	3 (75)	1 (25)	0	0
JSY n rural-21, n urban-22	17 (77.2)	5 (22.8)	18 (85.7)	3 (14.3)
ICDS n rural-32, n urban-51	40 (78.4)	11 (21.6)	22 (68.7)	10 (31.3)

ABHA is a randomly generated 14-digit number used for the purposes of uniquely identifying persons, authenticating them, and sharing their health records (only with their informed consent) across multiple systems and stakeholders. Findings in Table 4 highlight a notable disparity, with 10.2% of urban respondents reporting having an ABHA ID, compared to only 2.2% of respondents in rural areas. Overall, just 4.1% of all participants possessed an ABHA ID, revealing limited coverage and suggesting substantial gaps in awareness or accessibility, particularly for those living in rural regions.

**Table 4:** Distribution of respondents with ABHA ID by place of residence ABHA:  Ayushman Bharat Health Account

ABHA ID status	Urban (n = 3742)	Rural (n = 2900)	Total (n = 6642)
Yes	155 (4.1%)	115 (3.9%)	270 (4.1%)
No	3587 (95.9%)	2785 (96.1%)	6372 (95.9%)

Table 5 shows the various reasons for not having an ABHA ID among study participants. Aadhaar identity platform, with its inherent features of uniqueness, authentication, financial address, and e-KYC, enables the Government of India to directly reach residents of the country in the delivery of various subsidies, benefits, and services by using the resident’s Aadhaar number only [19]. Nearly half of the respondents reported that Aadhaar was available but not linked with their mobile (46.2%), which was slightly higher in urban areas (47.3%) compared to rural areas (46.4%). Lack of awareness about ABHA ID was the second most common barrier encountered by the urban residents. Overall, (17.1%) disproportionately affects urban residents (21.1% vs. 12.4% in rural areas). Mobile phone unavailability was another major constraint faced by residents of both locales. Similarly, the perception that ABHA ID has 'no utility' was more common in rural (9.5%) than in urban settings (4.4%). Other barriers, such as limited internet use, absence of Aadhaar, or lack of human support, showed lower overall prevalence but followed similar urban-rural gradients. This indicates that awareness and digital access gaps are the primary hurdles in rural populations, while perceived lack of utility dominates in urban contexts. 

**Table 5:** Reasons for not having an ABHA ID among study participants ABHA:  Ayushman Bharat Health Account

Reason for no ABHA ID	Total (n = 6372) (%)	Urban (n = 3587) (%)	Rural (n = 2785) (%)
Aadhaar not linked with mobile	2989 (46.23)	1697 (47.31)	1292 (46.39)
Aadhaar not available	375 (5.8)	176 (4.91)	199 (7.15)
Perceive no utility	424 (6.56)	158 (4.40)	266 (9.55)
Willing if usefulness explained	24 (0.37)	19 (0.53)	5 (0.18)
Internet unable to use	99 (1.53)	42 (1.17)	57 (2.05)
Lack of human assistance for generation	170 (2.63)	77 (2.15)	93 (3.34)
Mobile available, but no internet	262 (4.05)	143 (13.99)	119 (4.27)
Mobile phone not available	1020 (15.77)	517 (14.41)	409 (14.69)
Not aware of ABHA ID	1103 (17.06)	758 (21.13)	345 (12.39)

## Discussion

Health-care awareness and access gaps

A notable finding is the low level of health literacy regarding existing public health infrastructure; 50.3% of urban respondents and 55.0% of rural respondents reported no knowledge of existing health-care facilities. This lack of awareness persists even after major governmental efforts, like the National Rural Health Mission (NRHM), were launched to achieve faster and equitable improvements in maternal and child health outcomes by increasing accessibility and availability of care. Among those aware of services, knowledge is centred primarily on peripheral facilities like PHC/Wellness Centres (urban: 152; rural, 176) and CHCs (urban: 264; rural, 48). This data aligns with the broader challenges of unequal access to public health in India, which is influenced by economic status and rural-urban residence. The need for expansion of the healthcare system, especially in underprivileged and rural areas, is consistently noted in national dialogues addressing healthcare service gaps [16].

Utilisation of diagnostic services

The study revealed critically low utilisation of diagnostic services, with 97.4% of urban and 98.8% of rural participants reporting no utilisation of diagnostic tests in the last three months. This profound lack of usage highlights a major barrier to the early diagnosis and management of conditions, emphasising the rationale behind the primary Task Force study focusing on mobile diagnostic technologies.

Acceptability of doorstep services and privacy concerns

A central finding supporting the feasibility of the implementation research is the high and comparable acceptability of doorstep services across both settings, with 66.8% in urban and 66.6% in rural areas confirming acceptance. A similarly high rate was observed for the willingness to undergo doorstep blood tests. However, this positive finding must be balanced against the reported lack of comfort in sharing personal health status. A high percentage of respondents, 69.7% in urban areas and 68.3% in rural areas, were uncomfortable discussing their health status. This suggests that issues of privacy, confidentiality, and potentially stigma must be urgently addressed during implementation. Existing literature confirms that training health workers to address potential biases in their interactions based on socioeconomic status, class, and caste is critical for effective service delivery and reducing inequalities [5].

Role of CHWs

The study confirms the relevance of established frontline workers, with Anganwadi workers (53.1% urban, 48.5% rural) and Accredited Social Health Activists (ASHAs) (43.4% urban, 47.8% rural) being the most identified community workers. ASHAs are recognised under the NRM for their role in contacting pregnant women, facilitating institutional deliveries through behaviour change communication, and mobilising communities to use health facilities [5]. Given the high community recognition of these workers, they represent a critical human resource for increasing the uptake of new community-level diagnostic and screening services.

Limitations

As noted in the introduction to the Task Force study, the current data are limited by their cross-sectional nature, which prohibits causal inference. Furthermore, focusing on specific underprivileged populations in Bhopal sites means the results may not be generalisable to all rural and urban communities or geographical areas.

Recommendation

Implement doorstep diagnostic models to improve access and utilisation, particularly among underprivileged and rural populations. Establish strict confidentiality protocols and train frontline workers in ethical data handling to address community concerns regarding health information sharing. Conduct targeted Information, Education, and Communication (IEC) and Behaviour Change Communication campaigns via ASHAs and Anganwadi Workers to increase awareness and uptake of government health schemes beyond Ayushman Bharat and JSY. Organise local ABHA registration drives, simplify digital processes, and integrate enrolment within existing national programs to promote digital health inclusion.

## Conclusions

The situational analysis at the community level, carried out in urban and rural areas of Bhopal, demonstrated significant gaps in awareness, access, and use of health-care and diagnostic facilities. Knowledge about available health-care centers is suboptimal, and the use of diagnostic services is remarkably low. Encouragingly, most of the respondents showed strong support for doorstep health-care services, highlighting the potential for mobile diagnostic models to enhance primary health-care provision. Adoption of digital health, as indicated by the slow rate of ABHA ID registration, was yet another concern area, largely constrained by poor awareness. Overall, the report highlights the imperative to combine community-based diagnostic outreach, digital health, and health education in order to make substantial headway towards UHC. 

## Appendices

Project Title: A Task Force Study on Community-Level Situational Analysis in Urban and Rural Bhopal: Assessing the Availability and Acceptability for Doorstep Health-care Services

**Table 6:** Questionnaire

Section 1 : "Details of Project Sites”																					
101					Date of interview						DD/MM/YY										
102					Time of Interview						----/----										
103					Language of Interview						Hindi = 1, English = 2, Urdu = 3, Gujarati = 4							Assamese = 5, Kashmiri = 6, Tamil = 7, Others = 8			
104					Study Sites						Srinagar (J&K)) = 1, Assam (Guwahati) = 2, Bhopal (M.P) = 3, Varanasi (U.P) = 4							Muzzaffarpur (Bihar) = 5, Chennai (Tamil Nadu) = 6, ICMR Headquarter = 7			
105					Study Site Allocations						Urban Control = 1, Urban Intervention = 2, Rural Control = 3, Rural Intervention = 4										
106					Study Site Address						............................										
Section 2 : "Demographic profile”																					
201					Do you have ABHA No.						Yes=1 No=2										
a					If Yes, ABHA Number of Respondent (To be generated from NHA site)						......................... (14 digits only)										
b					If No, Reason for not Having ABHA No.						Mobile phone not available = 1, Mobile available but Internet not available = 2, Internet available but yet not able to use/ don’t know how to use internet = 3, Aadhar no. not available = 4, Aadhar no. available but not linked to mobile number = 5, Don’t find any use of getting ABHA Number = 6, Not willing to get ABHA Number = 7, Lack of human resources who can help in generating ABHA Number = 8, Electricity is not available frequently = 9, If any usefulness of ABHA number will be explained then may/will get ABHA number = 10, Will opt to get ABHA number if some health facility/ services is ensured = 11, Not aware of ABHA number = 12										
202					Any ID proof available?						Yes = 1, No = 2										
a					If Yes, ID proof?						Pan Number = 1, Aadhar Number = 2, Voter ID = 3, Driving licence = 4, Ration card number = 5										
b					If No, the person is giving consent for family to be a participant of the study						Yes = 1, No = 1										
203					Name of respondent						..........................										
204					Name of the head of the family						..........................										
205					ABHA no. of head of family						..........................										
206					Relationship of the respondent with the head of the family						Self (Head) = 1, Father = 2, Mother = 3, Son = 4, Daughter = 5, Wife = 6, Brother = 7, Sister = 8								Son in law daughter = 9, Daughter in law = 10, Grand children = 11, Sister-in law = 12, Niece = 13, Nephew = 14, Others = 15		
207					Address						.........................										
208					Contact Number						...........................										
209					Email (if any)						.............................										
210					Age (Completed years)						...........................										
211					Gender						Male = 1, Female = 2, Others = 3										
212					Duration of residence in that area (completed years)						By Birth = 1, 1-5 years = 2, 6-10 years = 3, Above 10 years = 4										
213					House Ownership						Owning House = 1, Rental = 2, Staying at Relative House = 3										
214					Family Type						Joint = 1, Nuclear = 2										
215					Marital Status						Never married = 1, Currently married = 2, Cohabitating /Living in Relation = 3, Separated = 4, Divorced = 5, Widow/widower = 6										
216					Religion						Hindu = 1, Muslim = 2, Christian = 3, Buddhism = 4, Jainism = 5, Sikh = 6, Other = 7										
Section 3: 'Socio-economic status of Family'																					
301					Total no. of Family members						........................										
302					Education						Illiterate = 1, Less than 8^th^ pass = 2, 8th pass = 3, 10th pass = 4, 12th pass = 5, Graduate = 6, Post graduate/professional degree = 7										
303					Occupation						Unemployed/home maker/ student-1 Unskilled worker-2 Semi skilled-3 Skilled-4										Clerk/ accountant-5 Semi professional-6 Professional-7
304					Total Monthly Income (Rs.)						..............										
305					Total Family Income per month (Rs.)						..............										
306					Land possession						no land =0 < 1 acre =1 1.5 acre =2 5-10 acre =3						10-15 acre =4 15-20 acre =5 >20 acre = 6				
307					House Possession-						Hut =1 Kutcha house =2 Mixed house =3, Pucca house =4, Mansion =5										
308					Farm power						No farm animals =1 1-2 farm animals =2 3-4 farm animals = 3 5-6 farm animals =4										
309					Material possessions						Bullock cart = 0 Cycle = 1 Two Wheeler=2 Three Wheeler=3 Four Wheeler=4 Radio =5 Laptop=6 Chairs =7							Mobile phone without internet =8 Mobile phone with internet=9 Television =10 Refrigerators =11 Others=12			
	Section 4 : "Eligibility Criteria for PMJAY”																				
	401	Eligibility for PMJAY (Tick if any criteria fulfilled) -				Residing in only one room with kuccha walls and kuccha roof =1 Landless households finding their income from manual casual labour =2 Female-headed households (where no adult male member between the age group of 16-59 years) =3 Disabled member and no able-bodied adult member =4 Scheduled Caste/Scheduled Tribe households =5 Households without shelter =6 Destitute/ living on alms =7 Manual scavenger families =8 Primitive tribal groups =9 Legally released bonded labour =10 Rag picker =11 Beggar =12 Domestic worker =13 Street vendor/ Cobbler/ hawker / Other service provider working on streets =14 Construction worker/ Plumber/ Mason/ Labour/ / Painter/ Welder/ Security guard =15 Coolie and another head-load worker =16 Sweeper/ Sanitation worker / Mali =17 Home-based worker/ Artisan / Handicrafts worker / Tailor =18 Transport worker/ Driver/ Conductor/ Helper to drivers and conductors/ Cart puller/ Rickshaw puller = 19 Shop worker / Assistant/ Peon in small establishment / Helper / Delivery assistant / Attendant / Waiter =20 Electrician / Mechanic/ Assembler/ Repair worker =21 Washer-man / Chowkidar =22															
	Section 5: "Physical Environment”																				
	501	Is there toilet within house?						Yes=1 No=2													
	a	If No, toilet what facility do you use?						Sulabh Public =1 Sharing with other households=2 Open defecation =3 Others = 4													
	502	How do you manage garbage?						Segregated =1 Mix all in one =2													
	503	Method of waste disposal						Giving it to waste collector=1 Dumping in community bin =2 Throwing somewhere else =3 Recycling =4													
	504	Type of fuel used						LPG =1 Electric =2 Biogas=3 Kerosene =4									Coal =5 Wood =6 Dung cakes =7 Others =8				
	505	Drinking water supply						Govt. Supply/ Tap water=1 Well/Tubewell/Boreholes=2 Hand pump =3												Bottled water =4 Rainwater/Pond/River =5	
	506	Method used to purify drinking water						No need to filter=1 Boiled =2 Filter =3 Seive =3													
	Section 6 : "Addictions Habits and Life style”																				
	601			Do you currently use any tobacco (smoke /smokeless) products						Yes=1 No=2 If No, GO TO Q.No. 602											
	a			If yes, what?						Cigarettes/bidi/Cigar =1 Toacco/ khaini =2 Paan/pan masala with Tobacco =3 Others =4											
	b			On an average trend in last one month for consumption of						aa) Khaini /tobacco(times per day) ............... bb) Paan/pan masala with Tobacco (times per day)..................... cc) Bidi/cigarettes/cigar (times per day)........... dd) Others drugs if any (times per day)............											
	602			Do you currently use any alcohol products						Yes=1 No=2 If No, GO TO Q.No. 603											
	a			If yes, the frequency of using alcohol?						Once a month or less =1 2-4 times a month =2 2-3 times a week =3 4 or more times a week =4											
	603			Are you a Vegetarian?						Yes=1 No =2											
	604			How many meals do you take in a day (no.)						.............................											
	605			Main ingredient of meal (tick multiple choice)						Dal =1 Chawal = 2 Roti=3 Sabji =4 Fruit =5 Fast /processed food =6											
	606			Type of sugar used,						White sugar (Chini) =1 Artificial sweetener =2 Brown sugar =3 Jaggary/gud =4, Khand/bura =5, Not taking sugar =6											
	607			Type of oil used in cooking						Ghee =1 Dalda =2 Refined oil =3 Mustard oil =4 others =5											
	608			Type of salt use						Kala namak =1 Sendha namak =2 Iodized salt =3 Non-iodized/sada =4 Not Taking Salt =5											
	Section : 7 "Physical Activities & Ailment History ”																				
	701			Physical activities (tick multiple choice) main activities during the day						Vigorous =1 Lifting load=2 Digging on construction=3 Sports=4 Yoga=5 Cycling/swimming=6 working for only self-care/bedridden self care + household work =7 self-care+ household + job with mild to moderate physical activity =8											
	702			History of ailment/disease in family-if any						No disease =1 High Blood Pressure/Hypertension =2 Diabetes/High blood sugar =3 High cholesterol =4 Any heart attack/chest pain/heart disease etc. =5 Kidney disease =6 Any other disease =7 If others specify.........................											
	Section 8 : "Laboratory Test facilities”																				
	801			Do you take regular health checkups/blood test from hospital or any private health facility								Yes=1 No=2 If No, GO TO Q.No-802									
	a			If yes, from where (Main source)?								Govt. Health facility=1 Pvt. Health facility=2 NGO/Trust=3									
	802			In recent times/ in last 3 months, how many times you or your family members have used laboratory facilities?								.........................									
	803			In recent times / in last 3 months, how much the lab test cost for family (in Rs.)?								.........................									
	804			Nearest Health Facility Distance (km) travelled for availing lab test?								0 km =1 1-5 km =2 >5 km =3									
	Section 9 : "Preferred Health Care system”																				
	901			Preference of Doctors?								Govt. Doctor =1 Private doctor =2 Both=3									
	902			Reason for going to visit these doctors?								Doctor is always available and ready to help =1 Cost is less/ economical health care =2 Trust in doctor/ faith/ belief =3 Feel that doctor have better skill and expertise =4 Fast and effective treatment - cost do not matter =5 Fast effective yet low cost treatment =6 Located nearby so time saved =7									
	903			Preference of medicine system?								Allopathy =1 Homeopathy=2 Ayurvedic=3 Unani=4 SIDDHA=5 Traditional =6 Others =7 =remove									
	904			Reason for choosing particular medicine system								Easily available =1 Cost is bearable =2 Trust/ faith/ believing that it is effective/ suits body =3 Fast and effective more useful =4 Effective, expensive =5 Effective/ also low in cost =6 Low in cost but effect is slow =7 Less side effects =8 Saves time =9									
	Section 10: "Available Government Health care facility”																				
	1001			Do you have knowledge of existing Government health facilities in your area?										Yes=1 No=2 If No, GO TO Q.No-1002							
	a			If yes, then, Type of health care facility?										PHC/Wellness Centre=1 CHC=2 District Hospital=3 FRC=4 Secondary Hospital=5 Tertiary Hospital=6							
	b			Distance of health facility from your residence (km)?										.............................							
	c			Fee charges by particular health facility/clinic/hospital cost ( Rs.)?										............................							
	1002			In last one year, number of hospital visit or any health centre by you/family member?										............................							
	1003			In last one year, Travel cost incurred for availing Government health services by the patient/family members (in Rs.)										............................							
	1004			In last one year Treatment cost (Approx in Rs.) paid by the patient/family members for availing Government Health Facilities?										...........................							
	Section 11 : "Information of existing/available health systems/ Tools / Mobile Application for collecting health data and Schemes”																				
	1101			Are you aware of any system - collecting health information? (disease-test treatment-cost- your needs elated information)												Yes=1 No=2					
	1102			Do you know about any mobile app for health education, health data uploading/consultation etc.												Yes=1 No=2 If No, GO TO Q.No. 1103					
	a			If Yes, have you downloaded any of these applications?												Yes=1 No=2					
	aa			If yes, Application name?												………………………					
	ab			How did you come to know about this health app?												Social media =1 Family member =2 By Neighbour =3 Friend =4 TV =5 Banner/ poster/ news papers = 6 Government health worker =7					
	ac			Who collects health app data?												........................					
	1103			Do you know that Govt. is creating a Unique Health ID for every individual?												Yes =1 No =2					
	1104			Do you have Unique Health ID?												Yes =1 No =2					
	1105			Do you know any Govt. scheme?												Yes =1 No =2 If No, GO TO Q.No.1201					
	a			If, Yes, which scheme?												.......................					
	a			Ayushman Bharat PradhanMantri Jan ArogyaYojana PMJAY												Yes =1 No =2 Not Applicable =3					
	b			If Yes, how much satisfied you are with this scheme?												Very satisfied=1 Satisfied=2 Normal=3 Dissatisfied=4 Very Dissatisfied=5					
	c			Pradhan Mantri Suraksha Bima Yojana PMSBY												Yes =1 No =2 Not Applicable =3					
	d			If Yes, how much satisfied you are with this scheme?												Very satisfied=1 Satisfied=2 Normal=3 Dissatisfied=4 Very Dissatisfied=5					
	e			Central Government Health Scheme CGHS												Yes =1 No =2 Not Applicable =3					
	f			If Yes, how much satisfied you are with this scheme?												Very satisfied=1 Satisfied=2 Normal=3 Dissatisfied=4 Very Dissatisfied=5					
	g			Employees State Insurance Scheme ESIC												Yes =1 No =2 Not Applicable =3					
	h			If Yes, how much satisfied you are with this scheme?												Very satisfied=1 Satisfied=2 Normal=3 Dissatisfied=4 Very Dissatisfied=5					
	i			Janani Suraksha Yojna												Yes =1 No =2 Not Applicable =3					
	j			If Yes, how much satisfied you are with this scheme?												Very satisfied=1 Satisfied=2 Normal=3 Dissatisfied=4 Very Dissatisfied=5					
	k			Anganwadi Yojana												Yes =1 No =2 Not Applicable =3					
	l			If Yes, how much satisfied you are with this scheme?												Very satisfied=1 Satisfied=2 Normal=3 Dissatisfied=4 Very Dissatisfied=5					
	m			State Health Insurance scheme (SHIS)												Yes =1 No =2 Not Applicable =3					
	n			If Yes, how much satisfied you are with this scheme?												Very satisfied=1 Satisfied=2 Normal=3 Dissatisfied=4 Very Dissatisfied=5					
	Section 12 : "Feedback on Door-Step Lab Health Facilities”																				
	1201			Would you like to get blood test done at your door step												Yes =1 No =2					
	1202			Is there any available lab facility for home based test?												Yes =1 No =2 If No, GO TO Q.No. 1203					
	a			If Yes, Then what does it cost for home based tests?												.......................					
	1203			In recent times/ in last 3 months, did you use any lab test facilities?												Yes =1 No =2 If No, GO TO Q.No. 1204					
	a			If yes, how many times you or your family members have used lab test facilities?												.........................					
	1204			Name all the Available lab test facilities in your nearby area? (List all the facilities)												All routine blood tests ( sugar hemoglobin, lft/kft) =1 X ray =2 CT =3 MRI =4 Urine =5 Stool =6 Specific biomarker/ advance tests =7 Don’t know = 8					
	1205			Are these facilities sufficient for your needs?												Yes =1 No =2					
	1206			Do you need any change in these lab test facilities?												Yes =1 No =2					
	1207			Does anyone has ever provided any health education to you or your family?												Yes =1 No =2 If No, GO TO Q.No.1208					
	a			If, Yes, Who provided health education?												..........................					
	b			How frequently that person (Health Educator) visits you? (Times)												Once a month =1 Twice a month =2 3 times a month =3 5 or more times a month =4 Available as and when required =5 Not available when required =6					
	1208			Does anyone has ever provided any routine screening facility to you or your family?												Yes =1 No =2 If No, GO TO Q.No.1301					
	a			If, Yes, Who provided routine screening facility?												............................					
	b			How frequently that person visits you? (times)												Daily =1 Weekly =2 Monthly =3 Yearly =4 Not available =5					
	Section 13 :"Feedback on Government Field Health Workers (ASHA/ANMs/Community Health Workers/ Anganwadi Workers) and proposed provision of Home Health Guide (HHG) ”																				
	1301			In recent times/ in last 3 months, how many times any Government Worker Visited you ?											........................						
	1302			Are you currently sharing your health data to the Government Health worker?											Yes =1 No =2 If No, GO TO Q.No.1303						
	a			If yes, with whom ?											Asha =1 ANMs =2 Anganwadi workers =3 Community workers =4 Others =5						
	1303			Would you be comfortable in sharing your health status with the Government health worker?											Yes =1 No =2						
	a			If No, Reason for not sharing health data with Government health worker											Not aware / no knowledge =1 Illiterate/ do not wish to understand =2 Don’t want to share to maintain secrecy =3 Nobody asks or use that data =4 No use of sharing health data =5 Not able to write by self- need help =6 If assistance given, benefit given- sure write and share data =7						
	1304			Would you be comfortable in sharing your health status in health diary?											Yes =1 No =2						
	a			If No, Reason for not sharing health data on health diary											……………………						
	1305			Would you be comfortable in sharing your health status on mobile application?											Yes =1 No =2						
	a			If No, reason for not sharing data on mobile application											……………………….						
	1306			Would you be comfortable to share/write your health problem in health diary by self?											Yes =1 No =2						
	a			If No, Reason for not sharing/writing your health problem in health diary by self?											……………………..						
	1307			Would you be comfortable to share/write your health problem online in Mobile application by self?											Yes =1 No =2						
	a			If No, Reason for not comfortable to share/write your health problem in mobile application by self?											Using simple phone- no feature to share =1 Illiterate/ not knowing how to share =2 Don’t think of any benefit- so will not share =3 If assistance is given- will share =4 No internet available =5 Don’t trust it will be used for service in return =6						
	1308			Do you need any help of a person (Health Guide) for filling your Health Problems in the Health Diary?											Yes =1 No =2						
	a			If No, Reason for no need of any help of a person (Health Guide) for filling your Health Problems in the Health Diary?											……………………..						
	1309			Do you agree if you share health data it will help to maintain health data /record at one place- so that it is easy for your doctor to know case fully, emergency care, get preventive health tips and proper guidance for treatment?											Yes =1 No =2						
	1310			What do you think how frequently health guide should visit you to screen you for disease and to update your health data ?											Once a month =1 2 times a month =2 3 times a month =3 Daily =4 Weekly =5 Yearly =6						
	Section 14 : "Feedback on recent Covid Pandemic”																				
	1401		Did any of your family member had covid?												Yes =1 No =2 If No, GO TO Q.No.1501						
	a		If yes, who?												......................						
	b		Age (in yrs)												......................						
	c		Gender												Male=1 Female=2 Others=3						
	d		Is treatment received?												Yes =1 No =2						
	dd		If Yes, Health facility giving treatment												Govt. Doctor =1 Private Doctor =2 Health worker =3						
	de		Who gave treatment												………………….						
	df		Treatment cost												………………….						
	dg		Experience												…………………						
	e		Was the patient Covid vaccinated?												Yes =1 No =2						
	Section 15 : “Anthropometric & Blood Test parameters”																				
	1501		Is Respondent Male / Female / Other?										Male =1 Female =2 Other =3								
	1502		Select criteria for male / female / Other :										Respondent is female (Non-Pregnant/Unmarried) =1 Respondent is female (Pregnant) =2 Respondent is male =3 Respondent is Other = 4								
	1503		Height (Adults)										......................... (cm)								
	1504		Weight										......................... (Kg)								
	1505		BMI										........................								
	1506		Waist circumference										........................								
	1507		Hip Circumference										........................								
	1508		Blood Pressure (BP)										.......................								
	Blood Test Parameters																				
	1509		Hemoglobin (Hb)										......................								
	1510		Blood Sugar (R)										......................								
	1511		Total Bilurubin										.....................								
	1512		Direct Bilurubin										.....................								
	1513		SGOT										....................								
	1514		SGPT										....................								
	1515		Alkaline Phosphatase (ALP)										....................								
	1516		Total Protein										....................								
	1517		Urea										....................								
	1518		Uric acid										....................								
	1519		Creatnine										....................								
	1520		HDL										....................								
	1521		LDL										....................								
	1522		Cholesterol										....................								
	1523		Triglyceride										....................								
	1524		Other Optional Test (On Demand) ECG:										.....................								
	Section 16 : "Details of Interviewer”																				
	1601			Name			.............................														
	1602			Designation			Social worker cum DEO=1 Lab Technician=2 Medical Officer/ Scientist=3														
	1603			Contact number			............................(10 digits only)														
	Section 17 : "Observations of Interviewer”																				
	1701			Was Household comfortable in sharing information?					Yes =1 No =2												
	a			If, No Reason for discomfort?					………………………..												
	1702			How did Interviewer found the respondent/ Household?					Co-operative =1 Non-Cooperative=2 Low knowledge about health=3 Adequate knowledge about health=4												
